# Neural Correlates of Outcome of the Psychotherapy Compared to Antidepressant Therapy in Anxiety and Depression Disorders: A Meta-Analysis

**DOI:** 10.3389/fpsyg.2017.00927

**Published:** 2017-06-07

**Authors:** Navkiran Kalsi, Daniela Altavilla, Renata Tambelli, Paola Aceto, Cristina Trentini, Chiara Di Giorgio, Carlo Lai

**Affiliations:** ^1^Department of Dynamic and Clinical Psychology, Sapienza University of RomeRome, Italy; ^2^Department of Anaesthesiology and Intensive Care, Università Cattolica del Sacro CuoreRome, Italy

**Keywords:** psychotherapy, pharmacotherapy, neural correlates, anxiety, depression

## Abstract

The most prevalent mental disorders, anxiety and depression, are commonly associated with structural and functional changes in the fronto-limbic brain areas. The clinical trials investigating patients with affective disorders showed different outcome to different treatments such as psychotherapy or pharmacotherapy. It is, however, still unexplored how these interventions approach affect the functional brain. This meta-analysis aims to compare the effects of psychotherapy compared to antidepressant therapy on functional brain activity in anxiety and depression disorders. Twenty-one samples with psychotherapy and seventeen samples with antidepressant therapy were included. The main finding showed an inverse effect of the two treatments on the right paracingulate activity. The patients undergoing psychotherapy showed an increase in the right paracingulate activity while pharmacological treatment led to a decrease of activation of this area. This finding seems to support the recent studies that hypothesize how psychotherapy, through the self-knowledge and the meaning processing, involves a top-down emotional regulation.

## Introduction

A human being is the outcome of a developing process, which depend on a complex interaction between the genetic information and the environment. A remarkable characteristic of the brain is that it allows the nervous system to process information from the interacting environment, modifying itself by experience in measurable ways (Markham and Greenough, 2004). With the recent advances in neuroimaging techniques, scientists are able to identify neural correlates not only of mental disorders but also of the changes associated with therapeutic interventions (Fuchs, 2004). These interventions are broadly categorized into psychotherapy or pharmaceutical treatments. However, it is very interesting to understand how the outcome of different treatments affect the functional brain activity and neural circuits (Salone et al., 2016).

The main impairment in affective disorders is related to emotional dysregulation and is characterized by abnormal brain activity in the cortico-limbic brain networks (Ochsner and Gross, 2008; Wager et al., 2008; Messina et al., 2013). Patients with depression showed hyperactivation of “default mode network” (DMN), consisting of the posterior cingulated, precuneus, inferior parietal lobule, medial prefrontal cortex, and of the amygdala during resting-state and in response to emotional stimuli (Greicius et al., 2007; Siegle et al., 2007; Grimm et al., 2009; Carlson et al., 2017). The patients with anxiety disorders showed multiple underlying structural abnormalities within the fear circuit, in particular of the ventromedial prefrontal cortex (Cha et al., 2014; Carlson et al., 2017) and an increased response in the amygdala, anterior cingulated cortex, and insula in anticipation of aversive and neutral stimuli (Stein et al., 2007; Nitschke et al., 2009; Carlson et al., 2011, 2017).

Accordingly, studies on the neurobiological outcomes of the therapeutic interventions in anxiety and depression disorders report the changes in neural activity in the cortico-limbic brain regions implicated in the emotion regulation (Ressler and Mayberg, 2007; Messina et al., 2013). With respect to prognosis and improvement of psychopathological symptoms, both psychotherapy and pharmacotherapy are clinically effective for treating psychopathological disorders (Cuijpers et al., 2013). Despite psychotherapy and pharmacotherapy seem to lead to a final common neurobiological pathway, it is reasonable to hypothesize that these widely differing treatments might engage diverse neural mechanisms (DeRubeis et al., 2008; Marano et al., 2012; Quidé et al., 2012). In according with this hypothesis, a recent meta-analysis showed that in patients with major depression the psychotherapy induced modifications in the left frontal, temporal, lingual gyri and in the cingulate cortex, as well as in the right frontal and precentral gyri. Otherwise, pharmacotherapy affected brain activation in the right insula (Boccia et al., 2016).

The pharmacotherapy is directly oriented to the emotional reactivity through the balance of neurotransmitter activity that seems to modify the neural activity in the limbic structures normalizing the cortical activity through bottom-up approach (Stahl, 2013). On the other side, the psychotherapy works on to build and to elaborate meanings that can regulate the attention and memory systems inducing changes in cortical brain activity that through top-down approach restores the limbic system functions reducing the emotional dysregulations (Linden, 2006).

Despite the impressive growth of neuroimaging techniques, how different treatments affect the functional brain is yet to be established.

In order to investigate the neural correlate of the outcome of different treatments used in the anxiety and depression clinical trials, in the present meta-analysis the therapeutic interventions were divided into two different categories: psychotherapy and antidepressant therapy.

The aim of the current meta-analysis was to compare the neural correlates of pre and post-treatment effects of psychotherapy and antidepressant therapy in affective disorders.

## Methods

In order to reach the aim of the meta-analysis, the following comparisons were performed: (1) pre- vs. post-treatment changes in the activations of brain regions due to psychotherapy (2) pre- vs. post-treatment changes in the activations of brain regions due to antidepressant therapy, and (3) post-treatment changes in the activations of brain regions due to psychotherapy vs. antidepressant therapy. Successively, the same contrasts only on resting state studies were re-performed. The significant effects for all contrasts were considered in both directions (increased and decreased effects post-treatment).

### Search criterion

A systematic search strategy was used to identify relevant neuroimaging studies reporting the changes in functional neural activity as a treatment outcome of cognitive/psychodynamic therapies and antidepressant therapy on the anxiety and depression disorders. For this purpose, PubMed and Scopus database search was performed by two independent researchers to find putative studies reporting the treatment for the anxiety and depression disorders following DSM-IV-TR criteria. The search was conducted for studies published between 2000 and 2016. The following search terms were used: “imaging,” “fMRI” (functional MRI), “PET” (positron emission tomography), and “SPECT” (single photon emission computed tomography) in combination with the name of the disorder (anxiety, PTSD, panic disorder, phobias, and depression). Furthermore, the reference lists of the articles were manually checked for the studies not identified in earlier literature search. To achieve a high standard of reporting we have adopted “Preferred Reporting Items for Systematic Reviews and Meta-Analyses” (PRISMA) guidelines.

### Selection criteria

Studies were included if they met the following criteria: (a) being original papers in a peer-reviewed journal, (b) involving subjects with a pre and post-treatment effects, (c) having employed functional imaging, (d) having reported the brain coordinates in standard brain atlases, (e) having adult samples (age 18–65).

The studies in which the entire group of patients had received prior interventions at the time of the start of the treatment study and the studies which reported a single case study were not included in the meta-analysis to reduce the possible biases.

### Recorded variables

The variables recorded for articles in the meta-analysis were: sample size, gender, mean age of participants and peak coordinates reported along with the software and stereotactic space of these coordinates. Additionally, we recorded the statistical significance of the treatment outcomes and the method employed to correct the results for multiple comparisons. The studies which compared between two types of treatments (psychotherapy vs. antidepressant therapy), only the pre to post brain changes for each treatment separately were considered.

### Study classification

The studies were then classified into two categories based on the treatment type. The studies that included the cognitive (CT) and dynamic (DPT) based treatments were categorized in psychotherapy group, and those including the intake of antidepressant drugs in antidepressant therapy group (see Table 1).

**Table 1:** Studies included in meta-analysis.

**Study**	**Disease**	**Therapy**	**Patients**	**Technique**	**Task**	**Mean age**
Farrow et al., 2005	PTSD	Psychotherapy	13	fMRI	Emotional task	42
Felmingham et al., 2007	PTSD	Psychotherapy	8	fMRI	Emotional task	36.8
Fu et al., 2008	Depression	Psychotherapy	16	fMRI	Emotional task	40
Huang et al., 2014	Depression	Psychotherapy	23	fMRI	Resting state	27.7
Kircher et al., 2013	Panic	Psychotherapy	42	fMRI	Emotional task	35.42
Paquette et al., 2003	Phobia	Psychotherapy	12	fMRI	Emotional task	24.8
Ritchey et al., 2011	Depression	Psychotherapy	11	fMRI	Emotional task	36.1
Sakai et al., 2006	Panic	Psychotherapy	11	PET	Resting state	29.8
Schienle et al., 2007	Phobia	Psychotherapy	14	fMRI	Emotional task	27.2
Yoshimura et al., 2014	Depression	Psychotherapy	21	fMRI	Emotional task	37.3
Aupperle et al., 2013	PTSD	Psychotherapy	14	fMRI	Emotional task	40.1
Buchheim et al., 2012	Depression	Psychotherapy	16	fMRI	Emotional task	38.9
Wiswede et al., 2014	Depression	Psychotherapy	13	fMRI	Emotional task	39.8
Beutel et al., 2010	Panic	Psychotherapy	15	fMRI	Emotional task	32
Furmark et al., 2002	Social Phobia	Psychotherapy; Antidepressant therapy	6; 6	PET	Emotional task	35.2
Goldapple et al., 2004	Depression	Psychotherapy; Antidepressant therapy	14; 13	PET	Resting state	41; 36
Kennedy et al., 2007	Depression	Psychotherapy; Antidepressant therapy	7; 9	PET	Resting state	32.7; 40.1
Konarski et al., 2009	Depression	Psychotherapy; Antidepressant therapy	7; 9	PET	Resting state	32.7; 40.1
Prasko et al., 2004	Panic	Psychotherapy; Antidepressant therapy	6; 6	PET	Resting state	31.8; 32
Brody et al., 2001	Depression	Psychotherapy; Antidepressant therapy	14; 10	PET	Resting state	40.7; 36.4
Martin et al., 2001	Depression	Psychotherapy; Antidepressant therapy	13; 15	SPECT	Resting state	38.4; 39.4
Brockmann et al., 2009	Depression	Antidepressant therapy	44	SPECT	Resting state	47.2
Carey et al., 2004	Anxiety	Antidepressant therapy	37	SPECT	Resting state	33.5
Kennedy et al., 2001	Depression	Antidepressant therapy	13	PET	Resting state	36.7
Mayberg et al., 2000	Depression	Antidepressant therapy	4	PET	Resting state	49
Samson et al., 2011	Depression	Antidepressant therapy	10	fMRI	Emotional task	41.5
Seedat et al., 2004	PTSD	Antidepressant therapy	11	SPECT	Resting state	33.6
Vlassenko et al., 2004	Depression	Antidepressant therapy	14	SPECT	Resting state	42.8
Warwick et al., 2006	Anxiety	Antidepressant therapy	31	SPECT	Resting state	33
Kilts et al., 2006	Anxiety	Antidepressant therapy	12	PET	Emotional task	38
Hoehn-Saric et al., 2004	Anxiety	Antidepressant therapy	6	fMRI	Emotional task	36

In these studies, the brain activity was scanned during the resting-state, focuses on spontaneous, low frequency fluctuations in the BOLD signal (Lee et al., 2013), or during an emotional task, i.e., during emotionally arousing stimuli (Messina et al., 2013).”

### Standard meta-analyses of functional changes post-treatment

Voxel-based meta-analyses of functional brain changes to the treatment were conducted with the effect-size version of signed differential mapping (ES-SDM; Radua and Mataix-Cols, 2009, 2012). This technique has been used in meta-analysis studies on obsessive compulsive disorder, schizophrenia and bipolar disorder, etc. (Bora et al., 2010, 2011; Palaniyappan et al., 2012). This method is based on using the peak coordinates to recreate, for each study, a map of the effect sizes of the differences between pre and post-treatment changes in patients, and then on conducting a standard random-effects variance-weighted meta-analysis in each voxel.

Between group comparison among the two treatments (psychotherapy vs. antidepressant therapy) was conducted and significant results were reported after threshold at *p* < 0.001 uncorrected (equivalent to *p* < 0.05 corrected for multiple comparisons (Radua et al., 2010) with an extent threshold of Ke > 10 voxels. Default ES-SDM kernel size and thresholds were used (FWHM = 20 mm, peak height Z = 1, cluster extent = 10 voxels; Radua and Mataix-Cols, 2009).

Robustness of the significant results was assessed by means of exploration of the jack-knife analyses by systematically repeating the meta-analyses by excluding one study at a time. If a significant brain region remains significant in all or most of the combinations of studies it can be concluded that this finding is highly replicable.

## Results

Thirty-one studies met the inclusion criteria. Four-teen studies tested neural correlate of psychotherapy (eleven using CT, three DPT) and ten studies investigated neural correlate of antidepressant therapy. The remaining seven studies were performed on two randomized trial (five with CT vs. antidepressant therapy and two with DPT vs. antidepressant therapy). Thus, the meta-analysis included 16 samples reporting treatment outcomes with CT, 5 samples with DPT and 17 samples with antidepressant therapy. The overall sample was equivalent to a cohort of 546 individuals undergoing treatment for anxiety and depression (Mean age, *SD* = 36.3, 5.46) contributing data to the meta-analysis.

### Comparison of regional brain response: psychotherapy and antidepressant therapy

Data for this analysis was obtained from 21 samples of 296 patients undergoing treatment with psychotherapy and 17 samples representing 250 patients undergoing antidepressant therapy.

As shown in Table 2, both psychotherapy and antidepressant therapy showed a decreased activation (post vs. pre) of the right inferior frontal gyrus, bilateral superior frontal gyrus, bilateral anterior cingulate, and right insula. However, other patterns of activations varied between these groups. In particular, patients undergoing psychotherapy showed an increased activation of right paracingulate gyrus and precuneus, and a decreased activation of right hippocampus, right parahippocampal gyrus, right amygdala, right rolandic operculum, right putamen, right temporal pole, right superior temporal gyrus, and bilateral anterior cingulate gyrus. Conversely, antidepressant therapy showed an increased activation (post vs. pre) in the right middle frontal gyrus, and a decreased activation of the bilateral supplementary motor area, bilateral paracingulate gyrus and bilateral caudate nucleus.

**Table 2:** Comparative results of treatment outcome of Psychotherapy and Antidepressant therapy.

	**Psychotherapy (Post** vs. **Pre)**					**Antidepressant therapy (Post vs. Pre)**					**Psychotherapy(Post-Pre)** vs. **Antidepressant therapy (Post-Pre)**									
											**Psychotherapy**					**Antidepressant therapy**				
		**Coordinates**	**SDM-Z**	***P*-value**	**Voxels**		**Coordinates**	**SDM-Z**	***P*-value**	**Voxels**		**Coordinates**	**SDM-Z**	***P*-value**	**Voxels**		**Coordinates**	**SDM-Z**	***P*-value**	**Voxels**
L lingual gyrus						↑	−12,−44,−2	1.19	0.00119	117										
L supramarginal gyrus						↑	−58,−28,28	1.04	0.00432	101										
L supplementary motor area						↓	−4,16,44	2.59	0.00002	367						↓	0,8,44	2.41	0.00001	307
							−4,12,48	2.41	0.00016	319							−4,2,44	1.78	0.00009	164
R supplementary motor area						↓	6,16,46	2.30	0.00011	224						↓	0,8,44	2.41	0.00001	169
							4,14,48	2.42	0.00012	186							4,2,46	1.78	0.0001	88
L inferior parietal						↑	−58,−28,28	1.04	0.00432	120										
L cerebellum						↑	12,−42,−16	1.17	0.00133	303										
R inferior frontal gyrus	↓	52,32,16	1.45	0.00121	300	↓	44,16,8	1.71	0.00169	155										
L middle frontal gyrus	↓	−34,54,−2	1.95	0.00015	280						↓	−34,54,−2	1.61	0.00040	187					
R middle frontal gyrus						↑	26,12,50	1.55	0.00017	124						↑	26,14,48	1.16	0.00140	48
							26,12,50	1.66	0.00012	123										
L superior frontal gyrus	↓	−8,42,−8	2.27	0.00001	699	↓	−4,18,40	2.47	0.00004	91	↓	−6,60,−4	1.61	0.00040	48					
		−6,60,−4	1.46	0.00165	254		4,14,48	2.42	0.00015	39										
R superior frontal gyrus	↓	8,42,−8	2.27	0.00001	582	↓	4,18,40	2.47	0.00004	75										
L paracingulate						↓	−6,10,42	2.51	0.00004	399						↓	−2,6,36	2.25	0.00002	452
R paracingulate	↑	14,−52,40	1.02	0.00054	119	↓	4,10,32	2.56	0.00003	337	↑	2,6,36	2.25	0.00002	306	↓	2,6,36	2.25	0.00002	306
L median paracingulate						↓	−8,14,38	2.07	0.00054	158						↓	−4,2,44	1.78	0.00010	319
R median paracingulate	↑	12,−12,38	1.16	0.00248	36	↓	8,10,38	2.04	0.00056	122	↑	4,2,34	1.48	0.00063	238	↓	4,2,34	1.48	0.00063	238
R hippocampus	↓	26,−4,−16	2.06	0.00002	84															
R parahippocampal gyrus	↓	26,−4,−16	2.06	0.00002	78															
R amygdala	↓	26,−4,−16	2.06	0.00002	193															
L anterior cingulate	↓	0,48,4	3.00	~0	674	↓	−2,8,36	2.41	0.00001	159										
		−2,38,2	1.33	0.00349	226															
R anterior cingulate	↓	0,48,4	3.00	~0	399	↓	2,8,36	2.41	0.00001	61										
R insula	↓	48,4,2	2.43	0.00001	702	↓	32,18,8	1.89	0.00074	265						↓	34,18,8	1.30	0.00230	51
							34,16,10	1.61	0.00305	25										
R rolandic operculum	↓	48,4,2	2.43	0.00001	305															
R Putamen	↓	48,4,2	2.43	0.00001	302															
R temporal pole	↓	48,4,2	2.43	0.00001	201															
R superior temporal gyrus	↓	48,4,2	2.43	0.00001	136															
R heschl gyrus	↓	48,4,2	2.43	0.00001	41															
L rectus gyrus	↓	−12,36,−10	2.23	0.00004	34															
L caudate nucleus						↓	−8,4,10	1.86	0.00082	17						↓	−10,8,16	1.36	0.00130	39
							−8,4,8	1.61	0.00308	15										
R precuneus	↑	14,−52,40	1.03	0.00054	86															

*SDM-Z: Voxel probability; threshold P-value: p = 0.005; Peak height threshold: z = 1. In red, results on only the resting-state studies*.

Interestingly, an inverse pattern of activation was observed in right paracingulate gyrus.

In order to control the effect of the task on the difference between outcome treatment of psychotherapy and antidepressant therapy, a comparison between the two treatments on only the resting state studies was conducted. Data for this analysis was obtained from 15 resting state studies representing 8 samples with 95 patients undergoing treatment with psychotherapy (25% anxiety and 75% depression disorders) and 11 samples with 192 patients undergoing antidepressant therapy (36% anxiety and 64% depression disorders). The findings confirm the inverse pattern of activation observed in right paracingulate gyrus in the previous analyses (Table 2).

### Robustness analysis

The analysis of robustness (jack-knife sensitivity analyses) showed that the results were highly replicable with possible exception of right parahippocampal gyrus, right superior frontal gyrus and bilateral caudate nucleus in antidepressant therapy group, and with possible exception of the left middle frontal activation in psychotherapy trials where this activation did not remain significant in 10/21 re-sampling combination trials.

## Discussion

This is to our knowledge is the first neuroimaging meta-analysis which focuses on a comparison between psychotherapy and antidepressant therapy in patients with anxiety and depression.

The patients undergoing psychotherapeutic and pharmacological treatments for anxiety and depression showed an overall decreased activations in right inferior frontal gyrus, bilateral superior frontal gyrus, bilateral anterior cingulate and right insula suggesting the relevant role of these areas in the symptoms reduction. Superior frontal gyrus and anterior cingulate represent the components of central executive networks of information processing which is activated when performing a task requiring focused attention. Furthermore, anterior cingulate and insula are involved in the Salience Network (Ham et al., 2013) which is responsible for switching between the default mode network (the network which is active during the rest when the brain is not engaged in a specific task) and the central executive network (Goulden et al., 2014). The intensity of interactions of default mode and central executive network in insular salience network activity have been previously associated with the severity of symptoms in major depressive disorder (Manoliu et al., 2013). The reduced post-treatment activations of these areas in anxiety and depression could indicate the restored activity of the brain.

The main finding of the present study was the inverse effects of psychotherapy and antidepressant therapy on the right paracingulate activity. The patients undergoing psychotherapeutic treatment led to an increased activation of the right paracingulate activity while those with antidepressant therapy showed a decrease of this area. The paracingulate cortex (approximately corresponding to BA32) is often considered to be part of the anterior cingulate cortex, however the BA32 has been described cytoarchitectonically as a cingulo-frontal transition area (Devinsky et al., 1995) and therefore anatomically (and maybe functionally) distinct from the anterior cingulated cortex (Gallagher and Frith, 2003). Specially, the paracingulate cortex activity seems to be associated with the mentalizing ability (Gallagher and Frith, 2003) and with self-monitoring such as: visual self-recognition (Kircher et al., 2000, 2001), autobiographical memory (Maguire and Mummery, 1999; Maguire, 2001), conflict monitoring (Botvinick et al., 2001; Beckmann et al., 2009), verbal self-monitoring (McGuire et al., 1996b), self-generated thoughts (McGuire et al., 1996a). The components of these abilities are implicated in initiation and maintenance of the symptoms of anxiety and depression (Roiser et al., 2012; Weightman et al., 2014; Anastasides et al., 2015; Bartczak and Bokus, 2015). Moreover, a study showed that the paracingulate cortex is active during the “rest” condition (Gusnard et al., 2001). The authors suggested that this might indicate a “default” mode of functioning in which “we think about ourselves” (Gusnard et al., 2001). In light of these findings, it seems that cingulo-frontal section could be specialized on internal mental states processing (Gallagher and Frith, 2003).

In a very interesting way, different authors reported that the anterior cingulated cortex is evolutionally very recent, in fact, it is present only in humans and in higher primates (Nimchinsky et al., 1999). Specifically paracingulate cortex seems to be present only in 50% of humans (Paus, 2001) and it might be indicative of a progressive evolution of this region in humans (Zilles et al., 1988; Gallagher and Frith, 2003), suggesting as its development could be affected by the environment and by the relative meaning attribution.

In line with the theoretical aspects of psychotherapies that emphasize the importance of the internal reality, as the representations, and the processes of the meaning attribution and elaboration (Timary et al., 2011), the increase of the right paracingulate activity might be interpreted as psychotherapy conditioned increase of the attention to personal inner states and of the emotions regulation ability (Keune et al., 2012; Messina et al., 2016).

Moreover, through the introspection and the self-knowledge, “a subject can construct itself as psychologically self-conscious (and not only as physically self-conscious) in an interplay of meta-representational abilities, autobiographical memory, and socio-communicative capacities” (Guerini et al., 2015). Conversely, the neurobiological outcome of the antidepressant therapy showed a decrease of the right paracingulate activity, which can be explained by the fact that this kind of treatment is not focused on the elaboration of internal mental states.

An alternative interpretation is that the inverse effect of the two types of treatment could be due to the different experimental tasks used during the neurobiological data acquisition (Messina et al., 2013). The results of the meta-analyses including only resting state studies, where the inverse effect of psychotherapy vs. antidepressant therapy on paracingulate activity was maintained, it falsifies this interpretation.

### Limitations

The present meta-analysis entails certain limitations. First, methodological limitation concerns the data available for analysis. Several studies had small sample sizes, variable duration, heterogeneity of techniques and study designs which might affect the outcome of the therapeutic outcome and thus the quality of the meta-analysis.

A second limitation is the considerable heterogeneity in the samples due to the combination of anxiety and depression together. However, in order to reduce the pathology biases, we considered homogeneous number of studies for anxiety and depression in psychotherapy and antidepressant therapy studies. Another limitation is that this meta-analysis also includes the studies reporting only the region of interest involving fronto-limbic brain. Finally, the present meta-analysis did not compare different types of psychotherapies (cognitive vs. dynamic). This lack was due to the exiguous number of clinical samples treated with dynamic psychotherapy. In order to perform this comparison more studies on the neurobiological outcome of dynamic treatment are needed.

## Conclusions

The finding of the present meta-analysis showed a different neurobiological outcome of the psychotherapy compared to antidepressant therapy in anxiety and depression. The psychotherapeutic and pharmacological treatments showed inverse effects on the right paracingulate activity. This finding seems to support the recent studies (Linden, 2006) that hypothesize how psychotherapy, through the self-knowledge and the meaning processing, involves a top-down emotional regulation.

## Author contributions

Participated in meta-analysis design: CL and NK. Performed data analysis: NK. Wrote or contributed to the writing of the manuscript: NK, DA, RT, PA, CT, CD, and CL.

### Conflict of interest statement

The authors declare that the research was conducted in the absence of any commercial or financial relationships that could be construed as a potential conflict of interest.
